# The Local and Systemic Exposure to Oxygen in Children With Severe Bronchiolitis on Invasive Mechanical Ventilation: A Retrospective Cohort Study

**DOI:** 10.1097/PCC.0000000000003130

**Published:** 2022-12-07

**Authors:** Thijs A. Lilien, Eleonore S. V. de Sonnaville, Job B. M. van Woensel, Reinout A. Bem

**Affiliations:** 1 Department of Pediatric Intensive Care Medicine, Emma Children’s Hospital, Amsterdam UMC location University of Amsterdam, Amsterdam, The Netherlands.

**Keywords:** critical care, hyperoxia, mechanical ventilation, oxygen/blood, oxygen/therapeutic use, pediatric intensive care units

## Abstract

Oxygen supplementation is a cornerstone treatment in critically ill children with bronchiolitis in the PICU. However, potential deleterious effects of high-dose oxygen are well-known. In this study, we aim to describe the pulmonary (local) and arterial (systemic) oxygen exposure over the duration of invasive mechanical ventilation (IMV) in children with severe bronchiolitis. Our secondary aim was to estimate potentially avoidable exposure to high-dose oxygen in these patients.

**DESIGN:**

Retrospective cohort study.

**SETTING:**

Single-center, tertiary-care PICU.

**PATIENTS:**

Children younger than 2 years old admitted to the PICU for severe bronchiolitis receiving IMV.

**INTERVENTIONS:**

None.

**MEASUREMENTS AND MAIN RESULTS:**

Hourly measurements of Fio_2_ and peripheral oxygen saturation (Spo_2_), and arterial blood gas data were collected up to day 10 of IMV. A total of 24,451 hours of IMV were observed in 176 patients (median age of 1.0 mo [interquartile range (IQR), 1.0–2.3 mo]). The pulmonary exposure to oxygen was highest during the first day of IMV (median time-weighted average [TWA]–Fio_2_ 0.46 [IQR, 0.39–0.53]), which significantly decreased over subsequent days. The systemic exposure to oxygen was relatively low, as severe hyperoxemia (TWA–Pao_2_ > 248 Torr [> 33 kPa]) was not observed. However, overuse of oxygen was common with 52.3% of patients (*n* = 92) having at least 1 day of possible excessive oxygen exposure and 14.8% (*n* = 26) with severe exposure. Furthermore, higher oxygen dosages correlated with increasing overuse of oxygen (r_repeated measures_, 0.59; 95% CI, 0.54–0.63). Additionally, caregivers were likely to keep Fio_2_ greater than or equal to 0.50 when Spo_2_ greater than or equal to 97%.

**CONCLUSIONS:**

Moderate to high-dose pulmonary oxygen exposure and potential overuse of oxygen were common in this cohort of severe bronchiolitis patients requiring IMV; however, this was not accompanied by a high systemic oxygen burden. Further studies are needed to determine optimal oxygenation targets to prevent overzealous use of oxygen in this vulnerable population.

Viral-induced bronchiolitis is among the most important injuries to the pulmonary system in early life (1, 2). Although generally a mild disease, children admitted to the PICU for severe bronchiolitis frequently require supplemental oxygen with invasive mechanical ventilation (IMV) to prevent and resolve life-threatening hypoxemia. However, toxic effects of prolonged high-dose oxygen exposure in adults and neonates have been well established (3, 4). Recently, a meta-analysis of studies in critically ill children found an association between a high systemic oxygen burden, as reflected by arterial hyperoxemia, and mortality (5). This further underlines the potential risk of oxygen treatment beyond the direct postnatal period. Consequently, in light of the pro-inflammatory pulmonary microenvironment associated with severe bronchiolitis (6), high-dose oxygen therapy may predispose these patients to a second injury.

The current practice of oxygen titration in children with (severe) bronchiolitis is still largely based on expert opinion, while international guidelines vary in their recommendation for oxygenation targets (7, 8). Better insight in the exposure to oxygen is needed to develop future study protocols on the potential role of oxygen toxicity in this vulnerable population in the PICU. In the current study, we aimed to assess the overall pulmonary (local) and arterial (systemic) exposure to oxygen in children with severe bronchiolitis receiving IMV. Our secondary aim was to estimate potentially avoidable exposure to high-dose oxygen.

## MATERIALS AND METHODS

This study was exempted by the Institutional Review Board of the Amsterdam UMC, location University of Amsterdam W22_127#22.168 (April 7, 2022).

Patients less than 2 years old who were admitted to the PICU, between December 2008 and December 2020, for severe bronchiolitis and required IMV were included.

Patient records were retrieved based at admission codes (**eTable 1**, http://links.lww.com/PCC/C278) and reviewed for eligibility. In brief, extracted data included: patient characteristics; outcome data; hourly nurse-validated ventilator data up to the 10th cumulative day of IMV; and results from arterial blood gas analyses (**eMethods**, http://links.lww.com/PCC/C278). Patients who were on IMV less than 24 hours, or more than 24 hours before admission, or those temporarily transferred to another PICU were excluded due to incomplete available data.

Fio_2_ and Pao_2_ were used to assess local and systemic oxygen exposure, respectively. Fio_2_ greater than or equal to 0.50 was defined as high, Fio_2_ 0.30–0.49 as moderate, and Fio_2_ 0.21–0.29 as low exposure (9). Pao_2_ greater than 100 Torr (> 13.3 kPa) was defined as supraphysiological and greater than 248 Torr (> 33 kPa) as severe hyperoxemia (5). To quantify possible excessive oxygen exposure, the cumulative excessive oxygen exposure score (CEE) was calculated (10). Oxygen administered above room air when peripheral oxygen saturation (Spo_2_) was greater than or equal to 97% was scored as excessive (11, 12). A sensitivity analysis was included with Spo_2_ greater than or equal to 95% (eMethods, http://links.lww.com/PCC/C278) (10). CEE of 0.17–0.25 was defined as high and greater than 0.25 as severe exposure (10). We also assessed the healthcare worker’s response on Fio_2_ settings if Spo_2_ was greater than or equal to 97% by assessing any change in Fio_2_ during the next hour of IMV.

For a comprehensive description of the statistical analysis, see eMethods (http://links.lww.com/PCC/C278). In summary, time-weighted averages (TWAs) were calculated per variable of interest for the duration of IMV (TWA_IMV_) and consecutive days of IMV (24-hr window [TWA_24h_]). Cases with substantial missing data were omitted. Ventilator-related data were assessed using a mixed-effects model. Fio_2_ and CEE score were evaluated using repeated measures correlation (13). Finally, we performed exploratory analyses on arterial line use and if TWA_24h_–Fio_2_ and TWA_24h_–CEE on the first day of IMV were associated with duration of IMV and length of stay (LOS) in the PICU. Analyses were performed using R 4.0.3 (R Foundation for Statistical Computing, Vienna, Austria) with RStudio 1.3.1093 (RStudio, Boston, MA).

## RESULTS

The selection of records is shown in **eFigure 1** (http://links.lww.com/PCC/C278). A total of 176 patients were included in the analysis. Descriptive statistics are reported in **eTable 2** (http://links.lww.com/PCC/C278).

A total of 24,451 hourly time points of IMV were observed, generating 987 TWA_24h_’s. A negligible proportion of observed time points lacked data (Spo_2_: *n* = 672, 2.7%; Fio_2_: *n* = 868, 3.5%). In total, 2,007 single Pao_2_ values could be extracted (546 TWA_24h_’s). Exploratory analysis showed that the use of arterial lines in our center depended at admission year (*p* < 0.001) and decreased over time (**eFig. 2**, http://links.lww.com/PCC/C278).

**Figure 1.**
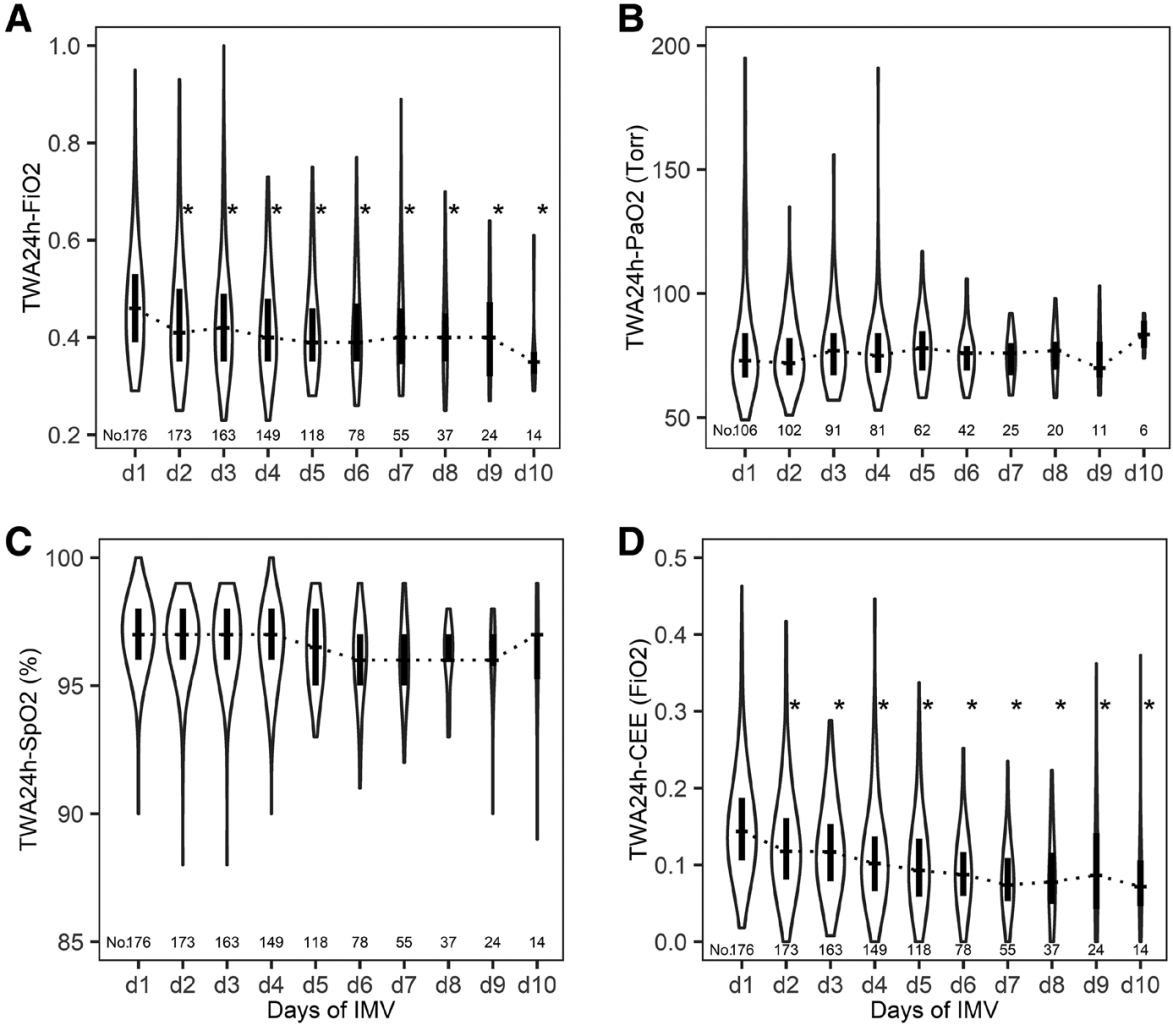
Time-weighted average per consecutive day (24 hr) (TWA_24h_) of invasive mechanical ventilation (IMV), TWAs were calculated by dividing the area under the variable by the observed time period. **A**, Fio_2_, **B**, Pao_2_, **C**, peripheral oxygen saturation (Spo_2_), **D**, cumulative excessive oxygen exposure score (CEE) (calculated by the Fio_2_ that was administered above room air [0.21], when oxygenation was sufficient, i.e., Spo_2_ ≥ 97%). *Dashed* and *black bars* represent the median and interquartile range, respectively. The *area* of the *violin plot* represents the density of individual data points scaled to the number of patients (No.) (displayed below *violin plot*). *p < 0.05 versus day 1 of IMV, calculated using a mixed-effects model.

Overall pulmonary oxygen exposure was moderate (median TWA_IMV_–Fio_2_ 0.41 [interquartile range (IQR) 0.36–0.47]) and overall systemic exposure was within physiologic limits (median TWA_IMV_–Pao_2_ 75 Torr [10.0 kPa] [IQR, 69–84]). In more detail, pulmonary exposure was highest on the first day of IMV (median TWA_24h_–Fio_2_ 0.46 [IQR, 0.39–0.53]) and significantly decreased during IMV (**Fig. 1**). However, short-term high-dose oxygen therapy was common among patients, with 89 patients (50.6%) having more than or equal to 1 day of high exposure, of which 25 patients (14.2%) had more than 3 days of high exposure (**Table 1**). Yet, none of these patients were exposed to severe hyperoxemia.

**TABLE 1.** Summary of Time-Weighted Averages per 24-Hour Window of Ventilator-Related Data

Variable (Time-Weighted Averages per 24-hr Window)	*n* Days^a^	*n* Subjects^b^, 1 d	*n* Subjects^b^, 2–3 d	*n* Subjects^b^, > 3 d	Total Subjects^b^, ≥ 1 d
Spo_2_, *n* (%)					
< 92%	9 (0.9)	3 (1.7)	2 (1.1)	0	5 (2.8)
Fio_2_, *n* (%)					
0.3–0.49	695 (70.4)	12 (6.8)	65 (36.9)	95 (54.0)	172 (97.7)
≥ 0.50	246 (24.9)	32 (18.2)	32 (18.2)	25 (14.2)	89 (50.6)
Pao_2_^c^, *n* (%)					
> 100 Torr (13.3 kPa)	38 (7.0)^d^	22 (15.7)^e^	7 (5.0)^e^	0	29 (20.7)^e^
> 248 Torr (33 kPa)	0	NA	NA	NA	0
CEE (Spo_2_ ≥ 97%)^f^, *n* (%)					
0.17–0.25	131 (13.3)	63 (35.8)	26 (14.8)	3 (1.7)	92 (52.3)
> 0.25	46 (4.7)	15 (8.5)	8 (4.5)	3 (1.7)	26 (14.8)
CEE (Spo_2_ ≥ 95%)^g^, *n* (%)					
0.17–0.25	283 (28.7)	52 (29.5)	68 (38.6)	16 (9.1)	136 (77.3)
> 0.25	174 (17.6)	41 (23.3)	27 (15.3)	15 (8.5)	83 (47.2)

CEE = cumulative excessive oxygen exposure score, NA = not applicable, Spo_2_ = peripheral oxygen saturation.

a Total observed days was 987.

b Total patients was 176.

c Data on Pao_2_ was only extracted from patients with an intra-arterial line.

d Total observed days was 546.

e Total patients with an intra-arterial line was 140.

f Primary threshold used to define overuse of oxygen.

g Threshold of sensitivity analysis to define overuse of oxygen.

Number of days and number of subjects that fulfill the different criteria used to categorize oxygenation, pulmonary (local), arterial (systemic), and excessive oxygen exposure, respectively.

Potential overuse of supplemental oxygen was frequently observed: 45.9% (*n* = 3,118) of time points with high-oxygen exposure were seen at Spo_2_ greater than or equal to 97%. Quantification of overall possible excess oxygen showed a median TWA_IMV_–CEE of 0.11 (IQR, 0.09–0.14). Possible excessive use was also highest on the first day of IMV (median TWA_24h_–CEE 0.14 [IQR, 0.11–0.19]) and significantly decreased during IMV (Fig. 1). Short-term high and severe possible excessive oxygen exposure were commonly observed (Table 1). Sensitivity analysis, using a lowered Spo_2_ threshold, substantially increased the proportion of patients exposed to possible excessive oxygen use (Table 1). Furthermore, use of higher oxygen doses correlated moderately to highly with increasing excessive oxygen exposure (**eFig. 3**, http://links.lww.com/PCC/C278). Additionally, in 50.6% (*n* = 1,531) of the observed time points that patients were exposed to high-dose oxygen and Spo_2_ was greater than or equal to 97%, the Fio_2_ was left unchanged during the next hour of IMV (**eFig. 4**, http://links.lww.com/PCC/C278).

Last, exploratory analysis showed an increase of TWA_24h–d1_–Fio_2_ was associated with an increase in median days on IMV and LOS in the PICU. We failed to observe this association for TWA_24h–d1_–CEE, except in sensitivity analysis, where TWA_24h–d1_–CEE_95%_ showed an association with an increase in median days LOS in the PICU (**eTable 3**, http://links.lww.com/PCC/C278).

## DISCUSSION

In this retrospective cohort of 176 children with severe bronchiolitis who received IMV, we found that the local exposure to oxygen was moderate to high in the majority of patients, whereas the systemic burden of oxygen was relatively low. Furthermore, in many cases, oxygen exposure was deemed potentially excessive and probably avoidable. We found that increasing oxygen dosage correlated with an increase in potential oxygen overuse.

The observed pulmonary oxygen exposure resembled the liberal oxygenation arm of the Oxygen in Pediatric Intensive Care (Oxy-PICU) pilot trial (14). Yet, this level of pulmonary exposure did not result in a high systemic burden, which is in line with studies in adult acute hypoxemic respiratory failure (15). This discrepancy in exposure is likely attributable to intrapulmonary shunting, as observed in acute respiratory distress syndrome, a common complication of severe bronchiolitis (16). This highlights that we should not focus solely on Pao_2_ to assess oxygen toxicity (5), as this marker fails to address potential adverse effects of high pulmonary exposure in patients with injured lungs (4, 9, 10, 17).

The observed tendency to treat patients with high-oxygen dosages was also observed in prior observational studies in critically ill adults (18, 19). Although avoiding overzealous oxygen exposure is an integral part of protective ventilation strategies, healthcare workers apparently experience difficulties following such recommendations (18, 19). Small-scale studies have demonstrated potential benefit of automated Fio_2_ closed-loop ventilation systems to improve Spo_2_ target adherence and reduce hyperoxia exposure (20, 21).

High-dose pulmonary exposure and overuse of oxygen have previously been associated with worse outcome independent of disease severity (10, 17, 22). This partially aligns with our findings, as initial Fio_2_ levels were associated with prolonged disease, but for possible overuse, we observed an association with outcome only in sensitivity analysis. However, it is important to emphasize that our study was not designed to analyze outcomes. Regarding the potential benefit of reducing oxygen exposure, a multitude of trials in critically ill adults and preterm neonates have not demonstrated a clear benefit of restrictive oxygenation targets so far (15, 23, 24). Furthermore, overly restrictive targets have even been identified as potentially dangerous in preterm neonates (24). The ongoing Oxy-PICU trial will provide first evidence on the effects, including on long-term outcomes, in children (25).

Strengths of this study were the comprehensive hourly data collection and exposure analysis. Limitations include: first, its retrospective design, which is inherently associated with selection bias and unaccounted confounders. This may have particularly affected the observed systemic exposure. Second, local protocols for oxygen titration may differ between centers, hindering the generalizability of our results. Third, albeit based on previous reports (10, 12, 18), in the absence of optimal Spo_2_ targets, our definition of excessive oxygen administration remains arbitrary. Fourth, this study was not primarily designed to analyze associations with outcome and our observations are therefore only of an exploratory nature.

## CONCLUSIONS

Moderate to high-dose pulmonary oxygen exposure and potential overuse of oxygen are common in children with severe bronchiolitis on IMV. As both of these have previously been associated with worse outcome in critically ill children, future studies on optimal oxygenation targets to reduce oxygen exposure are needed. In this regard, our findings help to develop study protocols for future trials in this field.
